# Statistical analysis of winning percentages in Japanese professional baseball using the Wins above Replacement indicator

**DOI:** 10.1371/journal.pone.0336297

**Published:** 2026-01-30

**Authors:** Shino Shimizu, Yasuhiro Suzuki

**Affiliations:** Graduate School of Informatics, Nagoya University, Nagoya, Japan; KAIST: Korea Advanced Institute of Science and Technology, KOREA, REPUBLIC OF

## Abstract

This study examines a previously unexplored concept in the field of sabermetrics. By employing a novel correction that accounts for player position differences, it investigates the impact of the designated hitter (DH) system on team wins in Japanese professional baseball using the Wins above Replacement metric. We applied this innovative correction to Pacific League data from 2014 to 2023, accounting for positional differences in player contributions. Our results indicate no significant difference in the correlation coefficients between team average wins above replacement (WAR) and winning percentage under conditions including and excluding the DH position, suggesting that the DH system does not substantially impact team performance. These findings offer a nuanced understanding of the DH system’s role in baseball strategy and team dynamics.

## 1. Background

### 1.1. History of sabermetrics

Over the past few decades, sabermetrics has emerged as a statistical approach for objectively analyzing baseball player performance. Pioneering works introduced concepts such as Linear Weights [1], which paved the way for metrics like the weighted On-Base Average (wOBA) [2] and comprehensive Wins above Replacement (WAR) statistics. WAR quantifies a player’s overall contribution to their team’s wins compared to a replacement-level player, making it valuable for evaluating players across different positions.

One rule that could influence WAR’s relationship with team success is the designated hitter (DH) system, introduced in 1973 in major league baseball’s (MLB) American League and in 1975 in Nippon Professional Baseball’s (NPB) Pacific League. The DH was aimed at boosting offensive output and fan interest by allowing a specialized hitter who does not field defensively. However, the strategic impact of this rule change and its broader effect on how individual talent translates to team performance remain unclear.

This study investigated how the presence of a DH influences the correlation between a team’s winning prowess (measured by WAR) and winning percentages in Japanese professional baseball. By comparing these correlations with and without the DH incorporated into the WAR calculations, we aim to provide insights into whether this rule substantially alters the balance between player skills and other factors, such as managerial strategy, in determining team success.

#### 1.1.1. Development of wOBA and WAR.

One of the key metrics used to calculate WAR is the weighted On-Base Average, developed by Tom Tango in 2007. wOBA measures a player’s overall offensive contributions per at-bat by assigning different weights to various offensive events (e.g., singles, doubles, and home runs) based on their run values. This approach provides a more nuanced understanding of a player’s offensive performance compared to traditional statistics such as batting average.

WAR is not a singularly defined metric; its calculation can vary depending on the data provider. However, it consistently aims to quantify the number of wins a player contributes to their team above what a replacement-level player would provide. This versatility makes WAR useful for comparing players across different positions and roles.

The DELTA method [3] used in this study incorporates Tom Tango’s wOBA into its hitting framework. By multiplying wOBA, an indicator of scoring contribution, by the number of at-bats, the process is enhanced for evaluating run creation.

### 1.2. Background of the DH system

Designated hitters do not play defense and focus solely on batting. This system did not exist from the inception of professional baseball in either the U.S. or Japan, having been introduced mainly for commercial reasons. Below is a brief background on the introduction of the DH in MLB and NPB. A simplified chronology is presented in Table 1 [4].

**Table 1:** Chronology of MLB and NPB.

MLB		NPB
Creation of the National League	1876	
Creation of the American League	1901	
	1948	Creation of Central and Pacific League
DH system introduced in the American League	1973	
	1975	DH system introduced in the Pacific League
DH system introduced in the National League	2022	

Note: MLB = Major League Baseball, NPB = Nippon Professional Baseball, DH = designated hitter.

The story of the MLB began in 1901 [4]. At this time, neither league had introduced the DH system. It was first implemented in 1973 by the American League. Low spectator attendance in the 1972 season was partly attributed to poor batting-to-pitching statistics, which made the games monotonous. Pitchers were limited to three or four hits per game, frustrating spectators who wanted more offensive action. The DH system was introduced to address this imbalance and help boost spectator attendance. The introduction of the DH system is credited with increasing the number of spectators in the American League [5]. In 2022, the DH system was adopted by the National League at the request of the Players Association. Currently, both MLB leagues have a DH system.

The history of the NPB begins in 1936 [4]. In 1948, the Central League and Pacific League, as they exist today, were formed. The DH system was first introduced in the Pacific League in 1975. A popular saying goes, “The Central League is popular, but the Pacific League is strong,” reflecting the greater popularity of the Central League [6] However, Pacific League ticket sales remained stagnant for a long period. To address this issue, the DH system was introduced in the Pacific League. Although the DH system has been discussed several times for the Central League, it has yet to be adopted, as noted in section 6.1.

As outlined above, the DH system was introduced for commercial reasons, specifically to increase the number of hits and thereby attract more spectators. Although the DH system offers several advantages for player success, it is interesting to note that this was not the primary motivation at the time of its introduction.

#### 1.2.1. Previous research and the DH system.

Studies on the impact of sabermetric indicators on team performance have produced mixed results. For example, Valero proposed a method for predicting MLB game outcomes using sabermetric statistics, achieving an accuracy of approximately 60% [7]. Beneventano et al. [8] evaluated whether sabermetric statistics are better predictors of run scores than traditional metrics and found that sabermetric indicators were superior in specific regression models.

The impact of the DH system on game outcomes and player performance has been debated within professional Japanese baseball. The DH system was introduced in the Pacific League in 1975 to increase offensive production and spectator interest. However, the Central League has not adopted a DH system, leading to ongoing discussions about its potential benefits and drawbacks.

Previous studies, such as those by Bradbury et al. [9] and Goff et al. [10], examined the effects of the DH system in MLB, suggesting that it can influence pitching strategies and potentially lead to more aggressive pitching, resulting in higher hit-by-pitch rates. Further, Kholodovsky et al. [11] also noted that the number of injuries to pitchers decreased with the introduction of the DH system, suggesting that the DH system also has an impact on the physical aspects of the players. It has also been reported that changes in player performance in response to rule changes, such as the introduction of the DH system, are remarkably similar in American and Japanese professional baseball [12].

### 1.3. Positioning of this study

This study aims to contribute to the ongoing discussion by examining the impact of the DH system on team success in the Pacific League from 2014 to 2023 using the WAR metric. As noted in the previous chapter, earlier studies have examined pitching effectiveness and player injury risk, but none have investigated the comprehensive metric of WAR in relation to team winning percentage [13–15]. Unlike previous studies, this research incorporates a novel correction that accounts for player position differences, offering a more refined analysis of the DH system’s impact on team performance. By comparing the correlation coefficients between team average WAR and winning percentage under two conditions—one including the DH position in the correction and the other excluding it—we sought to determine whether the DH system significantly influences team success. Compared to previous studies, the DH system is novel in that it can be evaluated using only a relatively simple formulation of sabermetric indicators without using complex data mining techniques.

This study uses data from Japanese professional baseball to explore the issue and demonstrate the DH system’s impact. The data were referenced from those published in 1.02 [3]. The methodology used here is broadly applicable. For instance, applying this research method to MLB data could evaluate the DH system’s impact on team success in the United States. Since there are no major differences in the operation of the DH systems between the two countries, similar results can be expected.

## 2. Outline of WAR

WAR is a comprehensive indicator that evaluates a player’s total contributions to their team, including batting, base running, and fielding, relative to a replacement-level player. While calculation methods for WAR may differ slightly between evaluators and data providers, the core concept remains consistent: quantifying a player’s overall impact on a team’s success.

- **Batting**: Metrics such as wOBA are used to quantify a player’s contribution to runs per at-bat.
- **Base Running**: Evaluates run creation through stolen bases and other base running actions.
- **Fielding**: Assesses defensive contributions by comparing a player’s performance to that of an average player at the same position, using metrics like the ultimate zone rating (UZR).
- **Positional Adjustment**: Accounts for differences in difficulty across defensive positions, allowing for fair comparisons between players in different positions.

### 2.1. Importance of regular correction

Regular correction is a novel approach introduced in this study to account for the value of players who consistently play without being substituted by reserve-level players. This correction adjusts the WAR calculation to reflect the player’s continuous contribution to the team’s performance, offering a more accurate measure than traditional replacement-level calculations.

This innovative use of regular correction by position aims to provide deeper insights into the relationship between the DH system and team success in professional baseball.

### 2.2. Terms and concepts

This section defines the terms and concepts used in this study.

Run creation: The number of runs scored by a player compared to the number of runs that would have been scored if a comparable player (e.g., a reserve-level player) had played in their place.

Win creation: The number of wins contributed by a player compared to the number of wins that would have been achieved if a comparable player (e.g., a reserve-level player) had played in their place.

Run expectancy: The expected number of runs scored by the end of an inning, given a specific situation (e.g., number of outs and base runners). This concept helps evaluate the impact of particular plays and strategies on run production.

Run value: The value assigned to specific plays (e.g., a single, double, or home run) based on their impact on run expectancy. It is calculated as the difference between run expectancies before and after the play. The run values used in this study are listed in Table 2.

**Table 2:** Run value in 2023.

Single hit	Double	Triple	Home run	Strike out	Base on balls	Caught stealing
0.414	0.740	1.072	1.397	−0.225	0.268	−0.368

Park factor (PF): A correction factor used to adjust for biases in player performance statistics caused by the unique characteristics of different ballparks. PF is calculated as the ratio of runs scored and allowed in a team’s home stadium to runs scored and allowed in other stadiums.

Regular correction: A novel approach used in this study to account for the value of players who consistently play without being substituted by reserve-level players. This correction adjusts the WAR calculation to reflect the continuous contributions of regular players, providing a more accurate measure of their impact than traditional replacement-level calculations.

### 2.3. WAR

The indicators used for WAR differ between fielders and pitchers. Additionally, the calculation method varies slightly depending on the company that handles the data. The following section introduces the calculation method used by DELTA [3]. As this study focuses primarily on the fielding WAR indicator, the calculation method is presented only for this indicator.

#### 2.3.1. Fielder’s WAR indicator.

The fielder’s WAR indicator is a framework that shows win creation for the subject player in a season compared to a hypothetical reserve-level player playing in the field.

The first step is to define the metrics used to establish the fielder’s WAR indicator. The fielder’s WAR uses five indicators: batting, replacement, base running, fielding, and positioning.

**Batting** Weighted On-Base Average: Defined as the contribution rate of runs per at-bat by the subject batter [3] (Equation (1)). Each coefficient is based on the run value. First, the absolute run value for an out is added to the run value of each play, setting the run value for an out to 0. Next, the wOBA scale is multiplied to correct the average wOBA to equal the on-base percentage.


$$\begin{array}{c} wOBA=\left\{\begin{array}{c} \text{0}.\text{6}\text{9}\text{2}\times (\text{walk}-\text{intentional}\;\text{walk})+\text{0}.\text{7}\text{3}\times \text{hit}\;\text{by}\;\text{pitch}\\ +\text{0}.\text{9}\text{6}\text{6}\times \text{reaching}\;\text{on}\;\text{an}\;\text{error}\\ +\text{0}.\text{8}\text{6}\text{5}\times \text{hit}+\text{1}.\text{3}\text{3}\text{6}\times \text{double}+\text{1}.\text{7}\text{2}\text{5}\times \text{triple}+\text{2}.\text{0}\text{6}\text{5}\times \text{home}\;\text{run} \end{array}\right\}\\ ÷(\text{at}\;\text{bat}+\text{base}\;\text{on}\;\text{balls}-\text{intentional}\;\text{walks}+\text{hit}\;\text{by}\;\text{pitch}+\text{sacrifice}\;\text{fly}) \end{array}$$ (1)

PF: This parameter is used to correct for bias in scores due to the shape and location of the ballpark. The PF correction value for the scores is given by Equation (2).


$$PF=\frac{\text{runs}\;\text{in}\;\text{home}\;\text{stadium}\;\text{per}\;\text{game}+\text{runs}\;\text{allowed}\;\text{in}\;\text{home}\;\text{stadium}\;\text{per}\;\text{game}}{\text{runs}\;\text{in}\;\text{away}\;\text{stadium}\;\text{per}\;\text{game}+\text{runs}\;\text{allowed}\;\text{in}\;\text{away}\;\text{stadium}\;\text{per}\;\text{game}}$$ (2)

For example, the Jingu Baseball Stadium, home of the Swallows, has a lower outfield fence than the Vantelin Dome Nagoya, home of the Dragons, making it easier to score home runs. As Table 3 shows, the Swallows are 1.17 times more likely to score runs than the league average, while the Dragons are 0.84 times more likely to do so. PF is used to counteract the effect of the team’s home stadium on players’ performance. The specific PF values for all teams are shown in Table 3 and Table 4. Batting represents run creation in a season by the subject hitter compared to the average hitter (Equation (3)). The average hitter is a hypothetical player with a wOBA equal to the average wOBA of all fielders who play on the first team each year. The hit rating is also called Weighted Runs above Average (wRAA) [3]. The wOBA scale is a constant used to adjust the wOBA and on-base percentage scales to match, and it varies in value from year to year.

**Table 3:** PF values in Central League.

T	C	De	G	Ys	D
0.99	0.95	1.03	1.02	1.17	0.84

Note: PF = Park factor.

**Table 4:** PF values in Pacific League.

Bs	M	H	E	L	F
0.94	1.09	1.03	0.93	0.99	0.96

Note: PF = Park factor.


$$\begin{array}{cc} Batting= & \;wRAA\;=\frac{wOBA\;\text{of}\;\text{the}\;\text{subject}\;\text{batter}\;\text{after}\;\text{PF}\;\text{correction}-wOBA\;\text{of}\;\text{league}\;\text{average}\;\text{fielder}}{wOBA\;scale}\;\\ & \times \text{plate}\;\text{appearance}\;\text{of}\;\text{the}\;\text{subject}\;\text{batter} \end{array}$$ (3)

**Replacement**: This represents the difference between the average and replacement levels during batting. The wOBA of the replacement-level hitter (reserve-level player) is defined as 0.88 times the wOBA of the average hitter, and the difference is taken (Equation (4)) [3].


$$\begin{array}{cc} Replacemant= & \;\frac{wOBA\;\text{of}\;\text{league}\;\text{average}\;\text{fielder}-\text{0}.\text{8}\text{8}\times wOBA\;\text{of}\;\text{league}\;\text{average}\;\text{fielder}}{wOBA\;scale}\;\\ & \times \text{plate}\;\text{appearance}\;\text{of}\;\text{the}\;\text{subject}\;\text{batter} \end{array}$$ (4)

Replacement is a framework for converting comparisons of batting from average hitters to reserve-level players. For base running and fielding, which are discussed below, reserve-level players are considered equal to average players; therefore, replacement is unnecessary.

Base running: Represents run creation by stolen bases and base running other than stolen bases. The difference in run creation compared with the average runner in the league is evaluated using stolen bases. The average runner is a hypothetical player with an average number of stolen bases and caught stealing among all players on the first team during the season. This is determined by comparing the subject player with the average runner in terms of run creation related to stolen bases and caught stealing. This is termed weighted stolen-base runs (wSB) and is expressed by Equation (5) [3].


wSB=A−B×C (5)

$$A=\text{steal}\times \text{stea}{\text{l}}^{\prime }\text{s}\;\text{run}\;\text{value}+\text{caught}\;\text{stealing}\times \text{caught}\;\text{stealin}{\text{g}}^{\prime }\text{s}\;\text{run}\;\text{value}$$ (5-1)

$$B=\frac{\left(\begin{array}{c} \text{all}\;\text{steal}\;\text{in}\;\text{league}\times \text{stea}{\text{l}}^{\prime }\text{s}\;\text{run}\;\text{value}\\ +\text{all}\;\text{caught}\;\text{stealing}\;\text{in}\;\text{league}\times \text{caught}\;\text{stealin}{\text{g}}^{\prime }\text{s}\;\text{run}\;\text{value} \end{array}\right)}{\left(\begin{array}{c} \text{all}\;\text{hit}\;\text{in}\;\text{league}+\text{all}\;\text{walk}\;\text{and}\;\text{hit}\;\text{by}\;\text{pitch}\;\text{in}\;\text{league}\\ -\text{all}\;\text{intentional}\;\text{walk}\;\text{in}\;\text{league} \end{array}\right)}$$ (5-2)

C=hit+walk and hit by pitch−intentional walk (5-3)

Run creation by base running, other than stolen bases, is also evaluated based on the difference in run creation compared with the average runner in the league. Base running other than stolen bases is defined as advancing bases on hits, breaking up a double play, or touching a base. Evaluators assign scores to each base running play, termed Ultimate Base Running (UBR). The value of UBR is published, but the calculation method is not. Base running is calculated by adding the run creation from stolen bases and from base running other than stolen bases (Equation (6)).


Base Runnning=wSB+UBR (6)

Fielding: Represents run creation through defense. It evaluates defensive opportunities by the number of runs prevented compared to an average fielder defending the same position [3].

The baseball field is divided into zones, and the zones where batted balls are hit and how they are handled individually are recorded. Subsequently, evaluations are made by comparing the average expected outs in each zone with the actual results of ball handling. This is termed ultimate zone rating (UZR). For all players except catchers, the UZR is used to calculate fielding. Although the value of the UZR is published, the calculation method is not. Additionally, UZR is not used for catchers because their primary role is not to handle batted balls; instead, they focus on catching pitches without deflecting them and preventing stolen bases.

Positional: This coefficient corrects for differences in UZR according to defensive position. As the UZR used for defensive evaluation is an indicator used for comparison within a defensive position, it does not consider differences in the difficulty of the position. Players in more difficult positions, such as catchers and outfielders, tend to possess higher defensive levels than players in other positions. Consequently, the average level is higher at more difficult defensive positions. This can result in lower UZR comparisons for players in such positions. The positional adjustment resolves this disparity in UZR across different defensive positions, allowing for fairer comparisons among players in various roles. Each defensive position is assigned a correction value, which is then multiplied by the number of innings pitched (Equation (7)) [3].


$$Positional=\text{fielding}\;\text{correction}\;\text{value}\times \frac{\text{fielding}\;\text{innings}}{\text{all}\;\text{fielding}\;\text{innings}\;\text{in}\;\text{team}}$$ (7)

The fielder’s WAR indicator is calculated based on the Runs above Replacement (RAR), which is the sum of the above components (Equation (8)).


RAR=Batting+Replacement+Base Running+Fielding+Positional (8)

RAR represents run creation in a season for a subject player compared to a reserve-level player. To convert RAR, an indicator of run creation, to WAR, an indicator of win creation, it is necessary to determine the relationship between wins and runs. This is expressed as runs per win (RPW). Thus, WAR and RAR have the relationship shown in Equation (9).


WAR[wins]=RAR[runs]÷RPW[runs/wins] (9)

## 3. Outline of regular correction

### 3.1. Definition of regular correction

As indicated in Section 2.3, WAR is an indicator based on reserve-level players. This is because reserve-level players, who act as substitutes when regular-level players are unavailable, are at a lower performance level than average players. Thus, it can be assumed that a regular player contributes to the team by playing an active role and keeping reserve-level players out of the game. Regular correction is an indicator that quantifies the value of regular player appearances [16]. This correction leads to a more accurate evaluation of a player who plays many games without relinquishing at-bats to reserve-level players.

Regular correction calculates the wRAA of reserve-level players for the number of at-bats played by regular players, as a reserve-level player is assumed to replace a regular player. Reserve-level players are defined as those who have participated in the first team even once, excluding those who regularly play on the first team. In other words, they are defined as those who are eligible to play on the first team but do not regularly play on the first team due to skill level or injury.

Given that the formula for determining wRAA is Equation (10), wRAA at the reserve level is given by Equation (11). This is derived by substituting the subject batter portion with a player at the reserve level.


$$\begin{array}{cc} wRAA= & \;\frac{wOBA\;\text{of}\;\text{the}\;\text{subject}\;\text{batter}\;-wOBA\;\text{of}\;\text{league}\;\text{average}\;\text{fielder}}{wOBA\;scale}\;\\ & \times \text{plate}\;\text{appearance}\;\text{of}\;\text{the}\;\text{subject}\;\text{batter} \end{array}$$ (10)

$$\begin{array}{cc} wRAA\;\text{at}\;\text{the}\;\text{reserve}\;\text{level}\;= & \;\frac{wOBA\;\text{of}\;\text{the}\;\text{reserve}\;\text{lebel}\;\text{batter}-wOBA\;\text{of}\;\text{league}\;\text{average}\;\text{fielder}}{wOBA\;scale}\;\\ & \times \text{plate}\;\text{appearance}\;\text{of}\;\text{the}\;\text{subject}\;\text{batter} \end{array}$$ (11)

When the reserve level is below the average level, the reserve-level wRAA is always negative. This means that if a reserve-level player continues to play in place of a regular player, the team’s score will be lower than that of an average hitter. Maintaining regular players in the game prevents a team’s scores from decreasing. Therefore, we define the regular correction as the reserve-level wRAA, which corresponds to the points that would have been lost had the reserve-level player played (Equation (12)). The regular correction is then added to the RAR of the regular player.


regular correction=−(wRAA at the reserve level) (12)

This adjustment allows for a higher RAR for players who regularly play many games, facilitating evaluation for their continued participation.

### 3.2. Significance of regular correction

**Fig 1:**
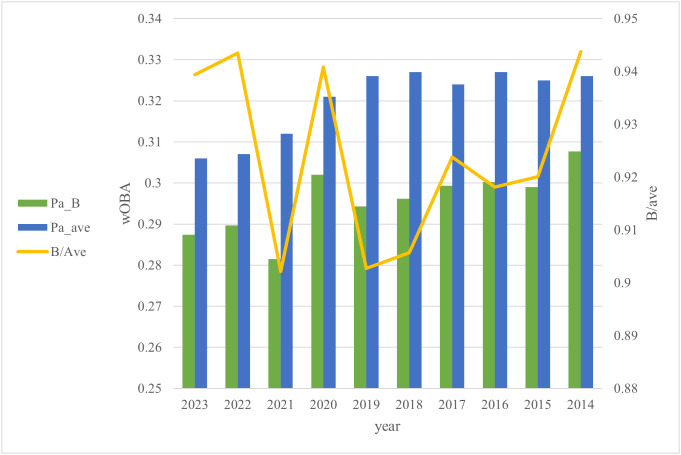
League average and reserve player weighted On-Base Average (wOBA) year ratios in the Pacific League. Pa_B: the wOBA of reserve players in the Pacific League. Pa_ave: the average wOBA of the Pacific League. B/ave: Pa_B/Pa_ave.

Consider a comparison with the replacement included in the framework to calculate the WAR adopted by DELTA [3]. Similar to regular correction, replacement is a framework that considers the gain from not allowing reserve-level players to play, based on the number of opportunities [3]. The intent of the calculation is similar to that of regular correction; however, the formulas differ (Equation (4)). Replacement defines the reserve-level wOBA as a theoretical value of 0.88 times the average. In contrast, in regular correction, reserve-level players are described as having the hitting level of non-regular players, with the value determined as a measured value. The average wOBA of regular players and league fielders changes over time, resulting in annual variation in the ratio. Fig 1 plots the year-to-year ratios of reserve players and league average wOBA in the Pacific League. Pa_B represents the wOBA of reserve players in the Pacific League, while Pa_ave represents the average wOBA of the Pacific League. The reserve players are all on the first team, except for the player with the most defensive innings during the season at each position. Fig 1 shows that the ratio of reserve-level players to league average wOBA varies widely each year, whereas replacement treats this ratio as constant across years. Thus, regular correction, which treats the reserve hitting level as measured, is superior for considering the gain from not allowing reserve-level players to play.

## 4. Methods

### 4.1. Outline of wRAA of reserve-level players by position

The traditional regular correction outlined in Section 3 calculates the wRAA for all reserve-level players, regardless of their defensive position. As Table 5 shows, regular catchers tend to have a lower wOBA because their leading and shoulder strengths are generally more critical than in hitting. In contrast, regular first basemen and left-fielders tend to have a higher wOBA because their strengths lie in hitting, making it less challenging to play defensively than in other positions. Considering the average wRAA for all fielders without accounting for position makes it impossible to evaluate the differences in batter levels by position, as described above. Therefore, it is more appropriate to calculate the wRAA of reserve-level players by position. This is defined as the wRAA of reserve-level players by position and is abbreviated as RP_wRAA. For this calculation, we consider Equation (13), where the subject batter in Equation (10) is replaced by reserve-level players by position:

**Table 5:** Average wOBA by position in Pacific League in 2023.

C	1B	2B	3B	SS	LF	CF	RF
0.280	0.317	0.302	0.305	0.294	0.322	0.299	0.308

Note: wOBA = weighted On-Base Average.


$$\text{RP}\_wRAA=\frac{\text{RP}\_wRAA-wOBA\;\text{of}\;\text{league}\;\text{average}\;\text{fielder}}{wOBA\;scale}\times \text{plate}\;\text{appearance}\;\text{of}\;\text{the}\;\text{subject}\;\text{batter}$$ (13)

This allows the regular correction to reflect differences in hitting by position. By calculating regular correction by position based on this RP_wRAA and incorporating regular correction by position into the RAR calculation framework, it is possible to calculate WAR that takes into account differences in blow levels by position.

### 4.2. Derivation of wRAA of reserve-level players by position

This study considers regular corrections in the Pacific League for the 10 years from 2014 to 2023 using RP_wRAA. In the NPB, the Pacific League is the only league that has introduced a DH system. To compare the difference in WAR with and without the DH system, this study used data exclusively from the Pacific League, where the DH system has been used. The player data for 2014–2023 is the maximum data available on the referenced data site [3] when the study was conducted. The following three rules are used when applying the regular correction:

1. The player eligible for regular correction is one at each position with the most defensive innings during the season.
2. The wRAA is a hitting indicator. As only fielders are considered, no regular correction is made for pitchers.
3. When calculating the wRAA, only the number of at-bats of the subject batter while in the subject defensive position is considered. This is not the total number of at-bats of the subject player.

For example, the total number of at-bats by Mannami (Fighters) in the 2023 season was 582, but the number of at-bats as a right fielder, his regular position, was 475. Since he also played first base and center field in addition to right field, the number differs from the total number of at-bats.

All data, such as batting average and wOBA, were obtained from DELTA’s comprehensive baseball data site named 1.02 [3]. Descriptive statistics for the data used are provided in Table 6. Some may argue that the idea of giving regular correction to only one player, as in 1, is problematic because it would cut down on the number of players below second place in a highly competitive position. However, in a highly competitive position, multiple players will compete for the opportunity to play, so the number of at-bats for which the player is eligible for regular correction will be reduced. Therefore, the number of opportunities to be discarded will also be smaller, and this is not considered to be a major problem. Furthermore, although selecting a single player could be viewed as an oversimplification of team dynamics, the adjustment through multiplication by the number of at-bats reduces this concern. In addition, the exclusion of pitcher WAR still limits the completeness of team performance analysis and, as noted later, remains a subject for further research.

**Table 6:** Descriptive statistics for the data used.

variables	average	standard deviation	25 percentile	median	75 percentile
wOBA in Pacific league	0.3201	0.008006	0.31075	0.3245	0.32625
total plate appearance in Pacific league	31922.9	1665.959	31866	32525.5	32823.5
total intentional base on balls in Pacific league	80.4	21.66195	64.75	75	100.25
total sacrifice hit on balls in Pacific league	636.2	116.833	544.75	597	728.5
wOBA scale	1.2818	0.033834	1.258	1.278	1.2985
wOBA of regular catcher	0.285383	0.04644	0.251	0.287	0.313
plate appearance of regular catcher	307.1	96.30294	236.25	297.5	367.5
intentional base on balls of regular catcher	0.416667	0.84245	0	0	1
sacrifice hit on balls of regular catcher	15.83333	8.554076	9.25	16	22
wOBA of regular first baseman	0.3438	0.035722	0.315	0.3385	0.36475
plate appearance of regular first baseman	348.8833	136.711	226.75	321	468.25
intentional base on balls of regular first baseman	1.366667	1.92325	0	0.5	2
sacrifice hit on balls of regular first baseman	1.55	2.801339	0	0	2
wOBA of regular second baseman	0.324183	0.034853	0.30325	0.323	0.3495
plate appearance of regular second baseman	406.2	157.4082	272.75	387	566.75
intentional base on balls of regular second baseman	0.816667	1.543715	0	0	1
sacrifice hit on balls of regular second baseman	7.45	7.802617	0.25	5	13
wOBA of regular third baseman	0.331783	0.034887	0.309	0.331	0.351
plate appearance of regular third baseman	369.5667	148.182	227	378.5	515.75
intentional base on balls of regular third baseman	0.916667	1.294754	0	0	1.75
sacrifice hit on balls of regular third baseman	2.15	3.434264	0	0	3
wOBA of regular shortstop	0.302667	0.031409	0.279	0.304	0.32275
plate appearance of regular shortstop	426.0667	135.2228	328.75	424	524.75
intentional base on balls of regular shortstop	0.283333	0.57999	0	0	0
sacrifice hit on balls of regular shortstop	18.5	13.02753	9.25	15.5	24.75
wOBA of regular left fielder	0.356	0.040575	0.33425	0.3555	0.37925
plate appearance of regular left fielder	346.5667	123.5446	235.25	350.5	433.75
intentional base on balls of regular left fielder	1.483333	2.224797	0	0	2
sacrifice hit on balls of regular left fielder	2.883333	3.966912	0	2	4
wOBA of regular center fielder	0.335017	0.060683	0.294	0.324	0.3735
plate appearance of regular center fielder	384.1333	164.7061	225.25	365	521.5
intentional base on balls of regular center fielder	0.966667	1.682921	0	0	1
sacrifice hit on balls of regular center fielder	5.883333	4.17968	3	6	9
wOBA of regular right fielder	0.339983	0.045952	0.307	0.3385	0.37025
plate appearance of regular right fielder	324.8667	121.2882	228	306.5	420.5
intentional base on balls of regular right fielder	1.45	1.75523	0	1	2
sacrifice hit on balls of regular right fielder	3.466667	4.649253	0	2	5.75
wOBA of regular DH	0.357167	0.044396	0.33075	0.3645	0.384
plate appearance of regular designated hitter	284.75	106.8402	206.75	261	349
intentional base on balls of regular DH	1.3	1.882374	0	1	2
sacrifice hit on balls of regular DH	0.233333	0.823947	0	0	0
wOBA of catcher in Pacific league	0.2804	0.009656	0.27075	0.28	0.28575
plate appearance of catcher in Pacific league	2952.9	156.1316	2947.5	3009.5	3026.5
intentional base on balls of catcher in Pacific league	3.4	1.68523	2	3.5	5
sacrifice hit on balls of catcher in Pacific league	156.5	29.0112	135.5	146	187
wOBA of first baseman in Pacific league	0.3317	0.011934	0.31925	0.332	0.3405
plate appearance of first baseman in Pacific league	3540	199.7709	3519.75	3610.5	3651.25
intentional base on balls of first baseman in Pacific league	10.9	4.482187	7.5	11.5	13.25
sacrifice hit on balls of first baseman in Pacific league	25.3	8.798295	19	24	36.25
wOBA of second baseman in Pacific league	0.3182	0.009453	0.3095	0.319	0.326
plate appearance of second baseman in Pacific league	3543.3	182.8191	3540	3592	3661
intentional base on balls of second baseman in Pacific league	6	2.408319	4	5.5	7.5
sacrifice hit on balls of second baseman in Pacific league	82.8	25.19841	68	74.5	89
wOBA of third baseman in Pacific league	0.3242	0.015689	0.30725	0.33	0.33525
plate appearance of third baseman in Pacific league	3467.4	181.7213	3464	3537.5	3556.75
intentional base on balls of third baseman in Pacific league	6.6	4.386342	3.75	4.5	8.5
sacrifice hit on balls of third baseman in Pacific league	37.6	13.49963	26.5	35	47.75
wOBA of shortstop in Pacific league	0.2969	0.007595	0.2915	0.297	0.30425
plate appearance of shortstop in Pacific league	3416.2	200.6319	3372.25	3479	3526
intentional base on balls of shortstop in Pacific league	1.9	1.374773	0.75	2	3.25
sacrifice hit on balls of shortstop in Pacific league	142	39.39543	109.25	119.5	181.25
wOBA of left fielder in Pacific league	0.3382	0.01227	0.32625	0.337	0.34825
plate appearance of left fielder in Pacific league	3560.5	178.5823	3540.5	3602.5	3663.5
intentional base on balls of left fielder in Pacific league	12.1	5.146844	8	12	16.25
sacrifice hit on balls of left fielder in Pacific league	39.7	8.198171	34.5	39	44.5
wOBA of center fielder in Pacific league	0.3302	0.020755	0.30725	0.3325	0.35125
plate appearance of center fielder in Pacific league	3580	213.1051	3517.5	3631	3740.5
intentional base on balls of center fielder in Pacific league	7.1	2.808914	4.75	7	10
sacrifice hit on balls of center fielder in Pacific league	68.6	12.24091	59.25	72.5	79.75
wOBA of right fielder in Pacific league	0.3265	0.012524	0.31325	0.3305	0.337
plate appearance of right fielder in Pacific league	3499	194.7403	3477	3546.5	3599.75
intentional base on balls of right fielder in Pacific league	12	3.768289	7.75	13	14
sacrifice hit on balls of right fielder in Pacific league	52.8	17.32513	36.75	50.5	71.75
wOBA of DH in Pacific league	0.3433	0.015047	0.3325	0.3395	0.357
plate appearance of DH in Pacific league	3301.6	108.8358	3273.25	3342.5	3368.75
intentional base on balls of DH in Pacific league	14.4	6.529931	9.25	14.5	18
sacrifice hit on balls of DH in Pacific league	8.2	4.422669	4	7.5	11.75
RPW	9.3858	0.325476	9.10825	9.4525	9.68175

From Equation (13), the following four data are needed to calculate the RP_wRAA.

1. wOBA of reserve level players by position (RP_wOBA)
2. fielder average wOBA by league
3. wOBA scale
4. subject batter’s at-bats

Data for points 2, 3, and 4 are available directly from DELTA’s site, named 1.02 [3], and are utilized. It is necessary to calculate the wOBA for point 1 because it is not mentioned in 1.02. Given that the formula for wOBA can be expressed by Equation (1), the wOBA of reserve-level players can be calculated using Equation (14). In the following formula, wOBA by position in league represents the wOBA of all players who played at each defensive position in the Pacific League. X by position in league represents the X of all players who played at each defensive position in the Pacific League.


$$\begin{array}{cc} wOBA= & \;\left\{\begin{array}{c} \text{0}.\text{6}\text{9}\text{2}\times (\text{walk}-\text{intentional}\;\text{walk})+\text{0}.\text{7}\text{3}\times \text{hit}\;\text{by}\;\text{pitch}\\ +\text{0}.\text{9}\text{6}\text{6}\times \text{reaching}\;\text{on}\;\text{an}\;\text{error}\\ +\text{0}.\text{8}\text{6}\text{5}\times \text{hit}+\text{1}.\text{3}\text{3}\text{6}\times \text{double}+\text{1}.\text{7}\text{2}\text{5}\times \text{triple}+\text{2}.\text{0}\text{6}\text{5}\times \text{home}\;\text{run} \end{array}\right\}\\ & ÷(\text{at}\;\text{bat}+\text{base}\;\text{on}\;\text{balls}-\text{intentional}\;\text{walks}+\text{hit}\;\text{by}\;\text{pitch}+\text{sacrifice}\;\text{fly}) \end{array}$$ (1)

$$\begin{array}{cc} \text{RP}\_wRAA= & \;\left\{\begin{array}{c} wOBA\;\text{by}\;\text{position}\;\text{in}\;\text{league}\times X\;\text{by}\;\text{position}\;\text{in}\;\text{league}\\ -\sum (wOBA\;\text{of}\;\text{the}\;\text{regular}\;\text{player}\times X\;\text{of}\;\text{the}\;\text{regular}\;\text{player}) \end{array}\right\}\;\\ & ÷(X\;\text{by}\;\text{position}\;\text{in}\;\text{league}-\sum X\;\text{of}\;\text{the}\;\text{regular}\;\text{player}) \end{array}$$ (14)

X=at bat+walk−intentional walk+hit by pitch+ sacrifice fly (14-1)

$$\begin{array}{cc} & \sum (wOBA\;\text{of}\;\text{the}\;\text{regularplayer}\times X\;\text{of}\;\text{the}\;\text{regular}\;\text{player})\;\\ & \begin{array}{c} =(wOBA\;\text{of}\;\text{Buffaloes}\;\text{regular}\;\text{player}\times X\;\text{of}\;\text{Buffaloes}\;\text{regular}\;\text{player})\\ +(wOBA\;\text{of}\;\text{Marines}\;\text{regular}\;\text{player}\times X\;\text{of}\;\text{Marines}\;\text{regular}\;\text{player})\\ \;\;\;\;\;\;\;\;\;\;\;\;\;\;\;\;\;\;\;\;\;\;\;\;\;\;\;+(wOBA\;\text{of}\;\text{Hawks}\;\text{regular}\;\text{player}\times X\;\text{of}\;\text{Hawks}\;\text{regular}\;\text{player})\\ +(wOBA\;\text{of}\;\text{Eagles}\;\text{regular}\;\text{player}\times X\;\text{of}\;\text{Eagles}\;\text{regular}\;\text{player})\\ +(wOBA\;\text{of}\;\text{Lions}\;\text{regular}\;\text{player}\times X\;\text{of}\;\text{Lions}\;\text{regular}\;\text{player})\\ \;\;\;\;\;\;\;\;\;\;\;\;\;\;\;\;\;\;\;+(wOBA\;\text{of}\;\text{Fighters}\;\text{regular}\;\text{player}\times X\;\text{of}\;\text{Fighters}\;\text{regular}\;\text{player}) \end{array} \end{array}$$ (14-2)

$$\begin{array}{cc} \sum X\;\text{of}\;\text{the}\;\text{regular}\;\text{player}\;= & \;X\;\text{of}\;\text{Buffaloes}\;\text{regular}\;\text{player}+X\;\text{of}\;\text{Marines}\;\text{regular}\;\text{player}\;\\ & +X\;\text{of}\;\text{Hawks}\;\text{regular}\;\text{player}+X\;\text{of}\;\text{Eagles}\;\text{regular}\;\text{player}\;\\ & +X\;\text{of}\;\text{Lions}\;\text{regular}\;\text{player}+X\;\text{of}\;\text{Fighters}\;\text{regular}\;\text{player} \end{array}$$ (14-3)

The detailed derivation of Equation (14) is described below.


$$\begin{array}{cc} wOBA= & \;\left\{\begin{array}{c} \text{0}.\text{6}\text{9}\text{2}\times (\text{walk}-\text{intentional}\;\text{walk})+\text{0}.\text{7}\text{3}\times \text{hit}\;\text{by}\;\text{pitch}\\ +\text{0}.\text{9}\text{6}\text{6}\times \text{reaching}\;\text{on}\;\text{an}\;\text{error}\\ +\text{0}.\text{8}\text{6}\text{5}\times \text{hit}+\text{1}.\text{3}\text{3}\text{6}\times \text{double}+\text{1}.\text{7}\text{2}\text{5}\times \text{triple}+\text{2}.\text{0}\text{6}\text{5}\times \text{home}\;\text{run} \end{array}\right\}\\ & ÷(\text{at}\;\text{bat}+\text{base}\;\text{on}\;\text{balls}-\text{intentional}\;\text{walks}+\text{hit}\;\text{by}\;\text{pitch}+\text{sacrifice}\;\text{fly}) \end{array}$$ (1)

$$\left\{\begin{array}{c} \text{0}.\text{6}\text{9}\text{2}\times (\text{walk}-\text{intentional}\;\text{walk})+\text{0}.\text{7}\text{3}\times \text{hit}\;\text{by}\;\text{pitch}\\ +\text{0}.\text{9}\text{6}\text{6}\times \text{reaching}\;\text{on}\;\text{an}\;\text{error}\\ +\text{0}.\text{8}\text{6}\text{5}\times \text{hit}+\text{1}.\text{3}\text{3}\text{6}\times \text{double}+\text{1}.\text{7}\text{2}\text{5}\times \text{triple}+\text{2}.\text{0}\text{6}\text{5}\times \text{home}\;\text{run} \end{array}\right\}÷X$$

$$wOBA\times X=\left\{\begin{array}{c} \text{0}.\text{6}\text{9}\text{2}\times (\text{walk}-\text{intentional}\;\text{walk})+\text{0}.\text{7}\text{3}\times \text{hit}\;\text{by}\;\text{pitch}\\ +\text{0}.\text{9}\text{6}\text{6}\times \text{reaching}\;\text{on}\;\text{an}\;\text{error}\\ +\text{0}.\text{8}\text{6}\text{5}\times \text{hit}+\text{1}.\text{3}\text{3}\text{6}\times \text{double}+\text{1}.\text{7}\text{2}\text{5}\times \text{triple}+\text{2}.\text{0}\text{6}\text{5}\times \text{home}\;\text{run} \end{array}\right\}$$ (1)ʹ

$$\begin{array}{cc} wOBA\;\text{by}\; & \text{position}\;\text{in}\;\text{league}\times X\;\text{by}\;\text{position}\;\text{in}\;\text{league}\\ & =\text{sum}\;\text{of}\left\{\begin{array}{c} \text{0}.\text{6}\text{9}\text{2}\times (\text{walk}-\text{intentional}\;\text{walk})+\text{0}.\text{7}\text{3}\times \text{hit}\;\text{by}\;\text{pitch}\\ +\text{0}.\text{9}\text{6}\text{6}\times \text{reaching}\;\text{on}\;\text{an}\;\text{error}\\ +\text{0}.\text{8}\text{6}\text{5}\times \text{hit}+\text{1}.\text{3}\text{3}\text{6}\times \text{double}+\text{1}.\text{7}\text{2}\text{5}\times \text{triple}+\text{2}.\text{0}\text{6}\text{5}\times \text{home}\;\text{run} \end{array}\right\} \end{array}$$

$$=\text{sum}\;\text{of}\left\{\begin{array}{c} \text{0}.\text{6}\text{9}\text{2}\times (\text{walk}-\text{intentional}\;\text{walk})+\text{0}.\text{7}\text{3}\times \text{hit}\;\text{by}\;\text{pitch}\\ +\text{0}.\text{9}\text{6}\text{6}\times \text{reaching}\;\text{on}\;\text{an}\;\text{error}\\ +\text{0}.\text{8}\text{6}\text{5}\times \text{hit}+\text{1}.\text{3}\text{3}\text{6}\times \text{double}+\text{1}.\text{7}\text{2}\text{5}\times \text{triple}+\text{2}.\text{0}\text{6}\text{5}\times \text{home}\;\text{run} \end{array}\right\}$$

$$\begin{array}{cc} \text{about}\;\text{all}\; & \text{reserve}\;\text{players}\;\text{by}\;\text{position}\;\text{in}\;\text{league}\\ & +\;\text{sum}\;\text{of}\left\{\begin{array}{c} \text{0}.\text{6}\text{9}\text{2}\times (\text{walk}-\text{intentional}\;\text{walk})+\text{0}.\text{7}\text{3}\times \text{hit}\;\text{by}\;\text{pitch}\\ +\text{0}.\text{9}\text{6}\text{6}\times \text{reaching}\;\text{on}\;\text{an}\;\text{error}\\ +\text{0}.\text{8}\text{6}\text{5}\times \text{hit}+\text{1}.\text{3}\text{3}\text{6}\times \text{double}+\text{1}.\text{7}\text{2}\text{5}\times \text{triple}+\text{2}.\text{0}\text{6}\text{5}\times \text{home}\;\text{run} \end{array}\right\} \end{array}$$

$$\begin{array}{cc} \text{about}\; & \text{all}\;\text{regular}\;\text{players}\;\text{by}\;\text{position}\;\text{in}\;\text{league}\;\\ & =\sum (\text{RP}\_wOBA\times X\;\text{of}\;\text{the}\;\text{reserve}\;\text{player})+\sum (wOBA\;\text{of}\;\text{the}\;\text{regular}\;\text{player}\\ & \;\;\times X\;\text{of}\;\text{the}\;\text{regular}\;\text{player})\;=\text{RP}\_wOBA\times (X\;\text{by}\;\text{position}\;\text{in}\;\text{league}-\sum X\;\text{of}\;\text{the}\;\text{regular}\;\text{player})\\ & =\text{RP}\_wOBA\times (X\;\text{by}\;\text{position}\;\text{in}\;\text{league}-\sum X\;\text{of}\;\text{the}\;\text{regular}\;\text{player})\\ & \;\;+\sum (wOBA\;\text{of}\;\text{the}\;\text{regular}\;\text{player}\times X\;\text{of}\;\text{the}\;\text{regular}\;\text{player}) \end{array}$$ (14)ʹ

$$\begin{array}{cc} \text{RP}\_wOBA\;= & \;\left\{\begin{array}{c} wOBA\;\text{by}\;\text{position}\;\text{in}\;\text{league}\times X\text{by}\;\text{position}\;\text{in}\;\text{league}\\ -\sum (wOBA\;\text{of}\;\text{the}\;\text{regular}\;\text{player}\times X\;\text{of}\;\;\text{the}\;\text{regular}\;\text{player}) \end{array}\right\}\;\\ & ÷(X\;\text{by}\;\text{position}\;\text{in}\;\text{league}-\sum X\;\text{of}\;\text{the}\;\text{regular}\;\text{player}) \end{array}$$ (15)

From Equation (14), the RP_wOBA is calculated. From the RP_wOBA, the RP_wRAA is calculated, and the regular correction by position is added to the RAR of the regular subject player. RAR after regular correction by position is defined as $\text{RA}{\text{R}}_{\text{2}}$. The process for deriving $\text{RA}{\text{R}}_{\text{2}}$ is shown in Equations (13), (16) and (17).


$$\text{RP}\_wRAA=\frac{\text{RP}\_wRAA-wOBA\;\text{of}\;\text{league}\;\text{average}\;\text{fielder}}{wOBA\;scale}\times \text{plate}\;\text{appearance}\;\text{of}\;\text{the}\;\text{subject}\;\text{batter}$$ (13)

$$\text{regular}\;\text{correction}\;\text{by}\;\text{position}=\left\{ \begin{array}{cc} -(\text{RP}\_\text{wRAA}) & \;\;(\text{RP}\_\text{wRAA}<\text{0})\\ \;\;\;\text{0} & \;\;(\text{RP}\_\text{wRAA}\geq \text{0}) \end{array}\right.\;$$ (16)

$$RAR_{\text{2}}=Batting+Replacement+Base\;Running+Fielding\;+Positional+regular\;correction\;by\;position$$ (17)

It should be noted that for Equation (16), RP_wRAA is favorable depending on the position. This is because RP_wOBA is a value that varies with position in Equation (13), whereas the average fielder’s wOBA by league is a constant that is independent of position. At a position where many players have advantages in hitting, the wOBA of reserve-level players is likely to be higher than the average wOBA of all fielders. This is because the hitting level is high, even at the reserve level. If the wRAA of the reserve level is positive, the regular correction value is negative. Even though they play regularly and contribute to the team, regular correction causes WAR to be smaller than it would be without regular correction. Since this is inappropriate, if the regular correction value is negative, the regular correction value is treated uniformly as 0. The positions and years in which regular correction was treated as 0 are shown in Table 7.

**Table 7:** Year and defensive position in which regular correction is 0.

	2023	2022	2021	2020	2019	2018	2017	2016	2015	2014
C										
1B						0	0	0	0	
2B		0								
3B										
SS										
LF		0		0						0
CF										
RF				0			0			
DH	0	0		0	0		0			0

For Equation (17), we added a regular correction to the conventional WAR framework, which allows us to extract and remove positions as shown in the next section.

### 4.3. Correlation between WAR and winning percentage

Using Section 4.2, we apply regular corrections to the RAR of regular players for the 10 years listed in 1.02 [3] and divide the RAR by the RPW to calculate the WAR. The following two patterns are considered to check for differences in the values with and without DH:

1. Cases in which DH is included in the positions subject to regular correction
2. Cases in which DH is not included in the positions subject to regular correction

Using the calculated WAR of all players, we take the average WAR of the players on each team for each year and use this as the team average WAR. The average here is the arithmetic mean.

We now consider the correlation coefficient between the average team WAR and team winning percentage. The winning percentage is defined by Equation (18). The games that resulted in draws are excluded.


$$\text{winning}\;\text{percentage}=\frac{\text{wins}}{\text{wins}+\text{wins}\;\text{allowed}}$$ (18)

## 5. Results

### 5.1. Correlation coefficient

**Fig 2:**
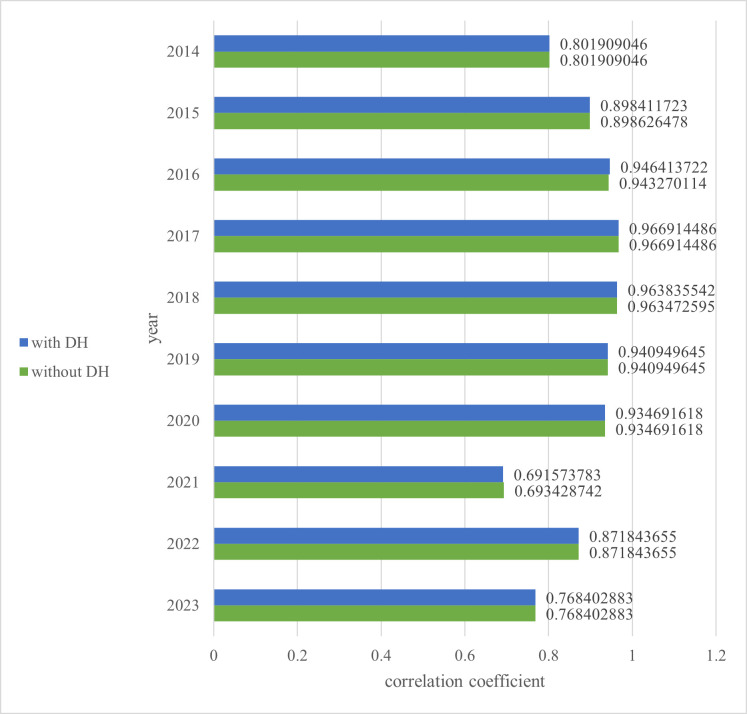
Correlation coefficient between WAR and team winning percentage in Pacific League.

The correlation coefficients are compared between the team winning percentage and WAR when the positions subject to regular correction include DH, and WAR when the positions subject to regular correction do not. In practice, this means a comparison of the Pacific League with and without the DH system in place. Although the DH system has been introduced, a comparison with the correlation coefficient in the case where it has not been introduced can lead to a consideration of its impact on team wins in practice. The results in Section 4.3 are shown in Fig 2 and Table 8, revealing that the correlation coefficients are consistent for some years. If RP_wRAA is positive in a given year, then regular DH players are given a regular correction of 0. In cases where the regular correction for DH is 0, the correlation coefficient does not change because there is no WAR change due to DH’s presence or absence. The correlation coefficients for 2014–2023 were approximately 0.001 lower for those with DH. We test whether this difference, including cases where the regular correction is non-zero, i.e., where there is a change in the correlation coefficient, is significant in the next section.

**Table 8:** Correlation coefficient between WAR and team winning percentage in Pacific League.

	2023	2022	2021	2020	2019	
With DH	0.7684029	0.8718437	0.6915738	0.9346916	0.9409496	
Without DH	0.7684029	0.8718437	0.6934287	0.9346916	0.9409496	
	2018	2017	2016	2015	2014	10-year total
With DH	0.9638355	0.9669145	0.9464137	0.8984117	0.8019090	0.8316733
Without DH	0.9634726	0.9669145	0.9432701	0.8986265	0.8019090	0.8320627

Note: WAR = Wins above Replacement, DH = designated hitter

### 5.2. Correlation coefficient difference test

We are testing for differences in correlation coefficients to confirm significant differences. The null hypothesis is that the two correlation coefficients are equal when the regular correction includes DH and when it does not. The alternative hypothesis is that the correlation coefficients are not equal. We define the required values from the 10 years of Pacific League data:

・$r_{\text{1}}=\text{0}.\text{831673313}$

: Correlation coefficient between WAR and team winning percentage, when regular corrections include DH

・$r_{\text{2}}=\text{0}.\text{832062675}$

: Correlation coefficient between WAR and team winning percentage, when regular corrections do not include DH

・$n_{\text{1}}=\text{6}\text{0}$

: Number of data points when the DH is included in the regular correction (six teams × 10 years)

・$n_{\text{2}}=\text{6}\text{0}$

: Number of data points when regular correction does not include the DH (six teams × 10 years)

Specific figs are taken from Section 5.1.

We apply Fisher’s z transform to $r_{\text{1}}$ and $r_{\text{2}}$ to approximate the known sampling distribution.


$$z_{\text{1}}=\frac{\text{1}}{\text{2}}\text{ln}\frac{\text{1}+r_{\text{1}}}{\text{1}-r_{\text{1}}}=\text{1}.\text{1}\text{9}\text{3}\text{5}\text{3}\text{9}\text{2}\text{7}\text{4}$$ (19-1)

$$z_{\text{2}}=\frac{\text{1}}{\text{2}}\text{ln}\frac{\text{1}+r_{\text{2}}}{\text{1}-r_{\text{2}}}=\text{1}.\text{1}\text{9}\text{4}\text{8}\text{0}\text{3}\text{4}\text{5}\text{5}$$ (19-2)

This is used to obtain the test statistic Z.


$$Z=\frac{z_{\text{1}}-z_{\text{2}}}{\sqrt{\frac{\text{1}}{n_{\text{1}}-\text{3}}+\frac{\text{1}}{n_{\text{2}}-\text{3}}}}=\frac{-\text{0}.\text{0}\text{0}\text{1}\text{2}\text{6}\text{4}\text{1}\text{8}\text{1}}{\sqrt{\frac{\text{2}}{\text{5}\text{7}}}}=-\text{0}.\text{0}\text{0}\text{6}\text{7}\text{4}\text{8}\text{8}\text{7}\text{9}$$ (20)

As Z follows a standard normal distribution, the probability ppp of being greater than or equal to |Z| is obtained from the standard normal distribution table. In this case, if the significance level α\alphaα is set to 0.05, the null hypothesis is not rejected when 2p>α. p=0.49601, since the significance level α=0.05, the null hypothesis is not rejected with 2p>α. In other words, no significant differences are observed between groups. Thus, we cannot conclude that the two correlation coefficients are not equal.

### 5.3. The impact of contextual factors

Contextual factors such as team strategy and coaching decisions are not taken into account in this calculation. The relevant factor that changes depending on such factors is plate appearance. This is because the opportunities for players with similar abilities to appear on the team vary depending on the coach’s decision and the depth of the team’s player roster. Therefore, we will consider contextual factors by examining the change in the correlation coefficient when the plate appearance of the subject batter in Equation (13) is changed.

Equation (13) was calculated by standardizing the plate appearances of the six players to the plate appearances of the player with the most/least plate appearances among the six players who are regulars at each defensive position (including DH) each year. The subsequent procedure is the same as in Section 4.3. The changes in the correlation coefficients resulting from this calculation are shown in Table 9. Clearly, there is no significant change, and we can conclude that contextual factors do not have a significant impact on the results of this study.

**Table 9:** Correlation coefficient between WAR and team winning percentage in Pacific League (max/min plate appearance version).

	2023	2022	2021	2020	2019	
Original	0.7684029	0.8718437	0.6915738	0.9346916	0.9409496	
Max	0.761699	0.87023	0.720665	0.910815	0.947717	
Min	0.769969	0.866762	0.708337	0.904385	0.954794	
	2018	2017	2016	2015	2014	10-year total
Original	0.9638355	0.9669145	0.9464137	0.8984117	0.8019090	0.8316733
Max	0.986052	0.966313	0.942146	0.892467	0.805888	0.809466
Min	0.988415	0.966557	0.936784	0.892718	0.809823	0.844501

Note: WAR = Wins above Replacement, DH = designated hitter

It is thus clear that the variation in opportunities to play due to coaching and team situation does not have a significant impact on the consideration of the impact of the DH system on team victories.

## 6. Discussion

### 6.1. Introduction of the DH system in the central league

As mentioned above, MLB introduced the DH system in both leagues, whereas the NPB has only introduced it in the Pacific League. This section discusses the advantages and disadvantages of introducing a DH system, based on the results of this study.

For the Pacific League, which already uses the DH system, we calculated the correlation coefficients between team average WAR and winning percentage in two patterns: with or without DH players included in the regular correction. WAR is a framework that shows the win contribution of a player in a season compared to a hypothetical reserve-level player. The higher the WAR, the more a player contributes to the team’s wins. Assuming team victories are based solely on player ability, teams with higher concentrations of players with higher WAR will have a greater winning percentage. However, team victories also involve non-player factors, such as the manager’s strategy. The higher the correlation coefficient, the more deeply a player’s ability is involved in winning. If the correlation coefficient is low, winning is primarily attributed to factors other than player ability. The DH system is one such factor. Therefore, if the correlation coefficient changes significantly when including or excluding DH players in the regular correction, the DH system is likely a significant factor in team victories. However, as discussed in Section 5.2, there were no significant differences in the correlation coefficients with or without the DH system. In other words, it cannot be said that the DH system affects the balance of player ability factors in team wins.

Although the DH system was introduced for commercial reasons, it also has potential advantages and disadvantages from both the players’ and fans’ perspectives. In 2022, MLB introduced the DH system in both leagues. In recent years, NPB has been debating whether this system should be introduced in the Central League. Regarding the burden on players, there is undoubtedly a difference between those playing with and without the DH system. It is presumed that the DH system would provide a significant advantage in offense because it would essentially replace the batter with a designated hitter instead of a pitcher. However, the results of this study indicate no direct impact on team wins. Some analysts believe that the DH system simplifies the manager’s role, allowing even less-skilled managers to win games if their players are good. However, based on this study’s findings, it does not follow that introducing the DH system will make a team’s success more dependent on players’ abilities. Moreover, if we follow the results of this study, it does not mean that the change in leadership due to the introduction of the DH system will make the players’ ability to win more reflected in their performance.

The DH system in Japan is used in most Pacific League games. Players with strong batting skills but weaker defense are often assigned as DH. Some teams consistently use the same player as DH, while others rotate multiple players in this role. Despite the DH system being in place, intended to simplify coaching and reduce player workload, the results of this study suggest no significant impact on team wins. The same can be said for Japanese high school baseball, which has not introduced a DH system.

The theoretical implication of the DH system is that it introduces significant changes to all aspects of baseball—hitting, pitching, and fielding—without affecting the magnitude of a player’s skill contribution to team victories, that is, without altering the overall game balance. Clarifying this background shows that the DH system does not fundamentally change the game but instead helps in understanding its impact from the perspectives of player evaluation and team strategy. Furthermore, as for the impact on international tournaments (MLB, KBO, and others that have already introduced the DH system), the results of this study can be used as material for consideration when the pros and cons of introducing the DH system are questioned again due to financial and other issues.

### 6.2. Comparison with conventional regular correction

In this study, regular corrections were made using RP_wRAA, which was designed to reflect differences in batter hitting levels by position. This section considers the differences between RP_wRAA and the conventional wRAA of reserve-level players.


$$\begin{array}{cc} wRAA\;\text{at}\;\text{the}\;\text{reserve}\;\text{level}\;= & \;\frac{wOBA\;\text{of}\;\text{the}\;\text{reserve}\;\text{lebel}\;\text{batter}-wOBA\;\text{of}\;\text{league}\;\text{average}\;\text{fielder}}{wOBA\;scale}\;\\ & \times \text{plate}\;\text{appearance}\;\text{of}\;\text{the}\;\text{subject}\;\text{batter} \end{array}$$ (11)

$$\text{RP}\_wRAA=\frac{\text{RP}\_wRAA-wOBA\;\text{of}\;\text{league}\;\text{average}\;\text{fielder}}{wOBA\;scale}\times \text{plate}\;\text{appearance}\;\text{of}\;\text{the}\;\text{subject}\;\text{batter}$$ (13)

**Fig 3:**
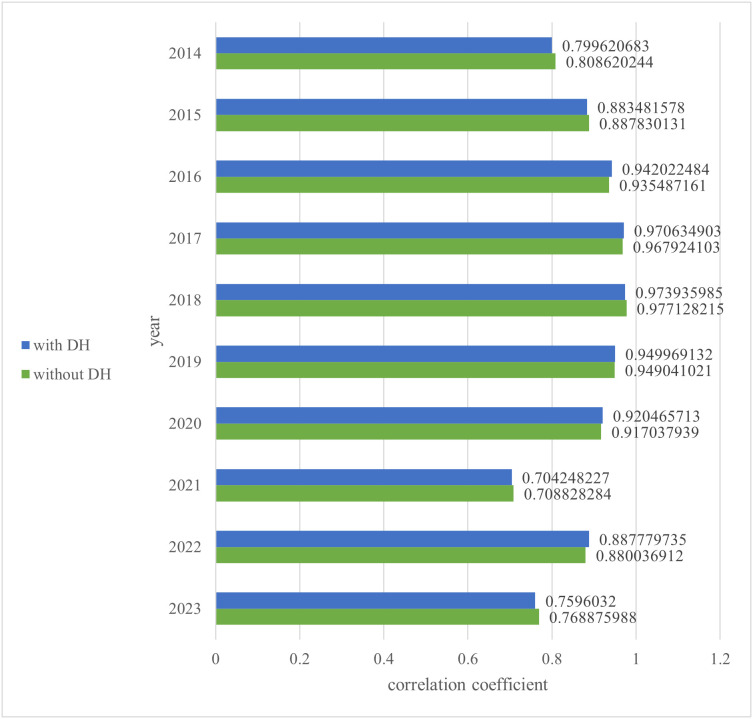
Correlation coefficient between WAR and team winning percentage in the Pacific League (Conventional Weighted Runs above Average version).

Equations (11) and (13) differ only in the first term of the fractional numerator (bolded). RP_wRAA uses RP_wOBA, which adjusts the reserve level for each position, unlike the conventional approach that applies the reserve level uniformly across all fielders. This positional adjustment does not alter the nature of wRAA when considering the correlation between WAR and winning percentage. To evaluate the effect of this adjustment, we recalculated the analysis from Section 4.3 using the conventional wRAA of reserve-level players, then compared the results with those obtained using RP_wRAA. The results using conventional wRAA are shown in Fig 3 and Table 10 alongside the correlation coefficient values from the regular correction by position. Tests were conducted to assess the difference in correlation coefficients for both the DH and non-DH cases, under conventional and positional regular corrections.

**Table 10:** Correlation coefficient between WAR and team winning percentage in the Pacific League (Conventional wRAA version).

	2023	2022	2021	2020	2019	
With DH	0.7596032	0.8877797	0.7042482	0.9204657	0.9499691	
Without DH	0.7688760	0.8800369	0.7088283	0.9170379	0.9490410	
	2018	2017	2016	2015	2014	10-year total
With DH	0.9439360	0.9706349	0.9420225	0.8834816	0.7996207	0.8444654
Without DH	0.9771282	0.9679241	0.9354872	0.8878301	0.8086202	0.8556799

Note: wRAA = Weighted Runs above Average, DH = designated hitter

Given the difference in how the wOBA of replacement-level batters is defined between RP_wRAA and conventional wRAA, there may be slight variations in actual values. However, the overall trend was expected to remain consistent.

The null hypothesis posits that the two correlation coefficients (from conventional and positional regular corrections) are equal, while the alternative hypothesis suggests they are not. For the DH case, the test yielded p=0.409046, and for the non-DH case, p=0.329969. In both cases, since 2*p* >α (with a significance level α=0.05), the null hypothesis was not rejected. Thus, no significant difference was observed, and it cannot be said that the two correlation coefficients are unequal. Therefore, the trends in the presence of the DH appear to be the same for both RP_wRAA and conventional wRAA. This suggests that RP_wRAA shares characteristics with the conventional wRAA for reserve-level players.

The advantage of RP_wRAA lies in its ability to reflect the different batting levels required for various positions. As shown in Table 11, positions such as catcher and ranger require stronger defensive skills, while positions like first baseman and left fielder prioritize batting ability. Consequently, the reserve-level skills required differ by position, making it more appropriate to consider wRAA by position when applying regular corrections.

**Table 11:** Replacement value by position in the 2023 season.

P	C	1B	2B	3B
60	18.1	−14.1	3.4	−4.8
SS	LF	CF	RF	DH
10.3	−12.0	4.2	−5.0	−15.1

One drawback of RP_wRAA is that it can yield values greater than zero. While the conventional wRAA is based on all fielder positions, making it unlikely that the reserve-level player’s wOBA would exceed the league average, RP_wRAA can exceed zero if the wOBA of the reserve player at a particular position is higher than the average league fielder’s wOBA. This can occur due to batting slumps among regular players or when players with stronger defensive skills (but weaker batting) are established as regulars. Applying a negative regular correction in these cases reduces a player’s WAR despite their regular contributions, which is inappropriate. To address this, the regular correction was uniformly set to zero when RP_wRAA exceeded zero. This is a known drawback of RP_wRAA.

**Fig 4:**
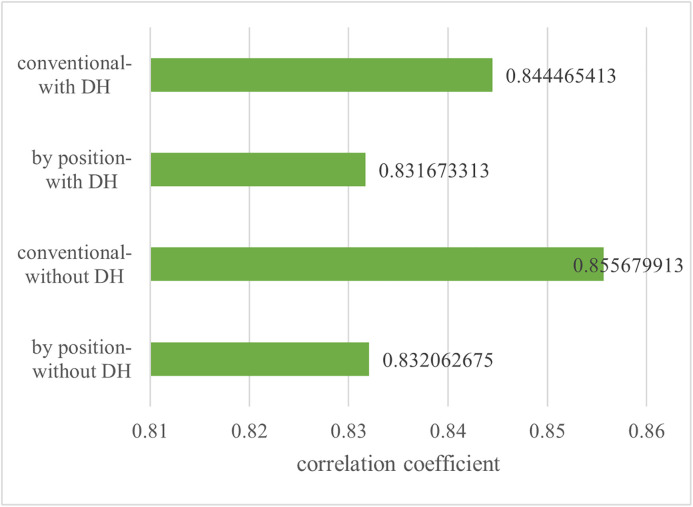
Correlation coefficient between average team WAR and team winning percentage (for 10 years).

Additionally, as shown in Fig 4, we compared the correlation coefficients between team average WAR and team winning percentage under both conventional and RP_wRAA-based regular corrections. The conventional correction yielded a higher correlation coefficient, indicating that accounting for reserve levels by position in RP_wRAA may lower the correlation between WAR and winning percentage.

### 6.3. Advantages of introducing regular corrections in this study

While replacement applies to all players in the first team, regular correction is applied to one player in each defensive position per team, making its scope of application narrower. This creates a clear distinction between regular players and other players. The evaluator determines which positions are subject to regular corrections. Therefore, as in this study, differences arising from the availability of the DH system could also be assessed. This was made possible by regular corrections, which cannot be achieved with replacement, as replacement requires calculations for all players.

Furthermore, compared to previous studies, the regular correction is superior in that it allows evaluation of the DH system using only relatively simple sabermetric indicator formulas without the need for complex data mining techniques.

## 7. Future research directions

The RP_wRAA used in this study was greater than 0 in positions requiring a high hitting level, which does not align with the purpose of regular correction—to accurately evaluate players who consistently play many games without yielding batting positions to reserve-level players. In such cases, regular corrections were treated as 0. However, given the aim of regular correction, this approach of assigning a value of 0 despite regular playing time is inadequate for optimal performance. Future research could explore ways to improve the wRAA to fully meet the objectives of regular correction.

**Fig 5:**
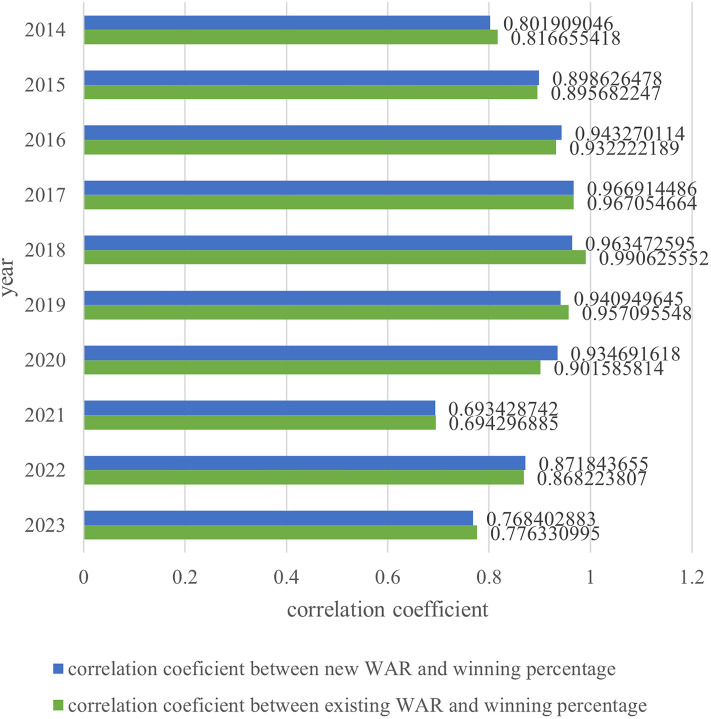
Correlation coefficient between WAR and team winning percentage in the Pacific League (Comparison between existing and new WAR).

As shown in Table 12 and Fig 5, the correlation coefficient between WAR and team winning percentage, calculated after applying regular correction, was lower than the correlation coefficient before applying regular correction. In this context, the WAR with regular corrections was used without DH. The larger the correlation coefficient between WAR and team winning percentage, the more significant the player’s contribution to winning. Although factors other than players, such as managerial strategy, may play a role, players should be the primary contributors to team success. Therefore, a future research direction could involve proposing a correction for WAR that would increase its correlation with team winning percentage.

**Table 12:** Correlation coefficient between WAR and team winning percentage.

	Correlation coefficient for 10 years
Without regular correction	0.865674079
With regular correction	0.855679913
With regular correction by position	0.832062675

Note: WAR = Wins above Replacement.

Prior research on modifying WAR has proposed an alternative indicator using weighted averages and principal component regression analysis with 13 different hitting indicators [17]. This study demonstrated a significant correlation between WAR and the proposed indicator, suggesting that the indicator is comparable to WAR. Future research could examine the possibility of developing a new indicator with a stronger correlation to team winning percentage, thereby compensating for limitations in the existing WAR. To this end, it would be useful to solve the aforementioned problem of the regular correction being zero, and to examine how the WAR index for pitchers, which was not the focus of this study, would change with the introduction of the DH system by changing the calculation method of tRA.

## 8. Conclusions

### 8.1. The DH system’s role in winning

This study compared the correlation coefficients between team-average WAR and winning percentage, both including and excluding DH players, in the regular correction calculation. The lack of a significant difference in these coefficients suggests that the DH system does not substantially alter the influence of players’ abilities on the team’s winning percentage. This finding implies that the commercially introduced DH rule does not significantly impact the balance between player skills and managerial strategies in determining game outcomes.

While the DH rule was initially introduced for commercial reasons, the results indicate that it does not directly affect the balance between player skills and other factors, such as managerial strategy, in deciding game outcomes.

If a significant difference had been found, it would suggest that the DH system changes the player-related factors affecting a team’s chances of winning. In this case, the practical aspect of the DH system is likely to be affected by the unique feature of the DH system, which forces the roster management to register more fielders. However, since no significant differences were observed in the correlation coefficients with or without the DH, the study concludes that the DH system does not influence team success. The change in the practical feature in the introduction of the DH system does not change the proportion of strategic impact on team victory. In practice, the adoption of the DH system is accelerating. Nevertheless, because the influence of player ability on team victories remains largely unchanged, managers are not required to make major strategic adjustments solely due to the presence or absence of the DH system.

### 8.2. Significance and limitations of the study

In this study, we applied regular corrections using the RP_wRAA. These corrections allowed us to reflect the differences in reserve levels by year and account for variations in player batting performance by position.

This methodology can also be applied to estimate how team performance might change if a DH system were introduced in the Central League. Although only Pacific League data were used, applying this approach to Central League data could help predict how the DH system would affect team performance and identify its advantages and disadvantages for both leagues in the NPB.

However, this study has several limitations. The impact of the DH system cannot be fully assessed by simply comparing correlation coefficients, and the RP_wRAA has some deficiencies. The statistical evaluation is limited, as it is difficult to establish causal relationships. While this study examined the correlation between the DH system and team winning percentage, other factors, such as player characteristics and stadium conditions in the Pacific League, may also have contributed to the results. Furthermore, pitcher WAR, which was not included in this study, may serve as a confounding factor. Although isolating such variables is challenging, future research should incorporate them into statistical models to more accurately evaluate the true impact of the DH system. Another limitation is the inability to verify robustness across different time periods, as our analysis relied on all available data from the referenced site. A central task for future research will be to define appropriate time periods while incorporating robustness checks.
